# FUNCTIONAL LIMITATIONS, DEPRESSIVE SYMPTOMS, AND COGNITION ACROSS MID- AND LATE-LIFE: EVIDENCE FROM CHINA

**DOI:** 10.1093/geroni/igae098.2653

**Published:** 2024-12-31

**Authors:** Chang Yu, Ashley Barr, Hanna Grol-Prokopczyk

**Affiliations:** University at Buffalo, SUNY, Buffalo, New York, United States; University at Buffalo, SUNY, Buffalo, New York, United States; University at Buffalo, SUNY, Buffalo, New York, United States

## Abstract

**Objectives:**

This study examines the time-varying effects of functional limitations (IADLs and ADLs) and depressive symptoms on various cognitive functions, including mental status and memory among middle-aged and older Chinese individuals. It also assesses whether functional limitations and depression during middle age contribute to worse cognitive function in the later years independent of yearly shifts in depression and functional limitations. Method: Using longitudinal data from the China Health and Retirement Longitudinal Study (2011-2018), we conducted mixed-effects linear models to determine the intra- and inter- individual variation on the relationship between functional limitations, depression, and cognitive functioning (N = 22,039 observations nested within 10,054 individuals).

**Results:**

Within-individual change in functional limitations and depression negatively predict cognitive functioning, with more functional limitations and depressive symptoms predicting a lower score in cognition, memory, and mental health, independent of age. Further, disabilities and depression in mid-life continue to predict worse cognitive functioning across the course of the study independent of any later changes in functional limitations and depressive symptoms and childhood conditions.

**Discussion:**

From a life course perspective, adverse functionalities and mental health in midlife predicts worse cognitive function in later life independent of early life experiences.

